# Gamification in dental education: Role of Kahoot in enhancing student engagement, learning outcomes and knowledge retention

**DOI:** 10.12669/pjms.42.9.16319

**Published:** 2026-09

**Authors:** Afsheen Mansoor, Fouzia Farrukh, Emaan Mansoor, Shabana Ali

**Affiliations:** 1 Afsheen Mansoor Associate Professor, Dental Material Sciences Department, Shaheed Zulfiqar Ali Bhutto Medical University, Islamabad, Pakistan; 2 Fouzia Farrukh Medical Education Department, Shaheed Zulfiqar Ali Bhutto Medical University, Islamabad, Pakistan; 3 Emaan Mansoor Islamic International Dental college, Riphah International University, Islamabad, Pakistan; 4 Shabana Ali Department of Medical Education, Riphah International University, Islamabad, Pakistan

**Keywords:** Dental education, Gamification, Game-based learning, Kahoot, Knowledge retention, Student engagement

## Abstract

**Objective:**

Digital technology in education has made a significant change in pedagogy where Gamification has become a novel approach of instruction involving competition, scoring, rewards and leaderboards that is capable of enhancing motivation and engagement of learners. Kahoot is the most widely used online game-based learning platform that not only create interactive quizzes but also conduct real-time testing which amplifies learning of educators. The objective of this narrative review was to assesses the effects of Kahoot in encouraging student engagement, enhancing learning and knowledge retention regarding dental education.

**Methodology:**

Literature search of major databases such as PubMed, Scopus, Web of Science and Google Scholar was carried out that focused on literature from 2020-2025. Keywords that were used in search included Kahoot, gamification, game-based learning, dental education and student engagement. Studies that investigated use of Kahoot or other gamified modes of learning in dental or in other contexts of health-profession education were included. Papers that did not include empirical evaluation or were not related to education were eliminated. First search brought about 312 records, upon eliminating duplicates, filtering titles and abstracts only. Seventy eight full-text articles were evaluated on their eligibility and 30 studies were finally selected as part of qualitative synthesis.

**Results:**

Synthesized evidence showed that classroom participation, active learning, motivation and engagement among students were improved with Kahoot. Various studies revealed significant positive changes in academic achievement and more conceptual level of understanding among participants. Kahoot supported formative assessment process because it depicted immediate instructor instant feedback that helped in identifying knowledge deficiencies during instructional process. Some drawbacks have been mentioned such as more reliance on technological infrastructure, lack of information regarding long-term results and non-utilization for summative assessment mechanisms.

**Conclusion:**

Kahoot is one of the most useful auxiliary learning tool in developing an interactive learning environment and enhancing retention of information for dental education. Further studies are required to determine its long-term effectiveness and overcome technological and pedagogical obstacles that can be encountered in its implementation.

## INTRODUCTION

The nature of dental education is in perpetual motion and both technological and pedagogical advancements should be introduced gradually which is the fundamental driving force of education involving both the stakeholders such as teachers and learners.^1^ Conventionally, the learning of dentistry has been greatly dependent on didactic lectures where teacher imparts knowledge in a passive way. Since years, this traditional paradigm has been in use as a single way of communication that does not serve the purpose of active engagement and critical learning deeply among the learners. Therefore, development of innovative methods of teaching for a two-way communication between teachers and learners in order to promote engagement, critical thinking, active learning, peer interaction, and collaborative understanding always remained a challenge. The only solution to address this situation has been the invention of novel and interactive digital technology-based tools commonly referred as gamification.^2^

The Gamification involves the integration of unique game-based features including points, rewards, competition, leaderboards etc. into educational context to make the learning process more engaging and motivating to learners. The induction these gamification elements would enrich the classroom activities by converting the classical lecture-based teaching into more active and engaging learning process. The use of Gamification in the health professions education field has become the most widespread option for enhancing the student’s involvement altogether with their academic performances. Since, dental education is a highly qualified paradigm where learners need to integrate intricate theoretical knowledge with realistic clinical skills. Therefore, it has become essential to implement current pedagogical methods that might be play an active role in both knowledge gaining and learning on the larger scale.^3^

Gamification learning tools provide the students with a chance to be actively involved in the process of not only covering the course content but also cementing it through practice. Among the numerous gamification platforms, Kahoot has become popular due to its several up to the mark characteristics such as: easy-to-use interface, wide accessibility, fun giving and fluid interactivity. The platform allows the instructor to design quizzes and surveys that students can answer by using smart phones, tablets, laptops or computers in real time and space. It has a well-defined competitive system with the provision of instant feedback and scoring system that makes the learning process more interesting, captivating, motivating with the elements of fun and enjoyment. Current literature stated that Kahoot have posed substantial positive influence on the classroom engagement and satisfaction of the students.^4^

The comparison of Gamified activities to conventional lectures revealed that game oriented educational tools like quizzes are more entertaining which enhances a greater level of motivation and perseverance during the learning process.^5^ In addition, Kahoot is an effective tool of formative assessment that enables instructors to track the understanding level of students pertaining the curriculum in real-time.^2^,^3^ The use of gamification in the field of education has been addressed to some extent but it is still a developing topic of interest in the dental education that needs to be unveiled to the best of our knowledge. The impact of Kahoot on student engagement, performance, learning outcome and knowledge retention attributed to dental education needs to be addressed on the larger extent. Therefore, this narrative review is of great importance as it aimed to investigate the role of Kahoot in dental training and critically analyzed the existing evidence on its effectiveness as a gamified learning tool.

### Concept of gamification in education:

The utilization of Gamification in education refers to the inclusion of the digital oriented quiz to teach the learners. In this way, teachers can strive to increase the interest and intrinsic motivation of learners. The general goal of gamification in educational context is to foster the interactive learning environments enabling active learning and consequently, enhancing the teaching results. The unique characteristics of gamified instructional design are point allocation systems, leaderboards, challenges that have limits in time, physical rewards like badges, and the immediate, formative feedback.^6^ These are the mechanisms that are used to encourage the curiosity and the competition of the learners, therefore cementing their motivation and participation. The effectiveness of gamification is supported by a number of proven learning theories of which constructivist theory is the most important one which leads to the belief that learners create knowledge in participatory and experiential learning activities.^7^ Thus, Gamified activities motivate students to develop relationships with the content as well as work with their co-workers, eventually reaching a more advanced and subtle understanding.^8^

### Overview of Kahoot as a gamified learning tool:

Kahoot is an online learning tool that can be used as an example of gamified learning implemented into face-to-face as well as distance educational settings. This platform allows the educator to create interactive quizzes and educational exercises, where the learners are asked to answer on their personal devices and the questions are on a common screen at the same time. The main properties of Kahoot include live testing, grading features, functionality features, real-time feedback, multimedia compatibility, and wide accessibility on smartphones and computers. All this created a competitive and collaborative environment that supports the attention of students during the delivery of the lecture.^9^ The ease of navigation and overall high level of customizability make the platform an appropriate tool in a very broad range of academic environments, including both undergraduate and post-graduate dental programs (Fig.1).

**Fig.1:**
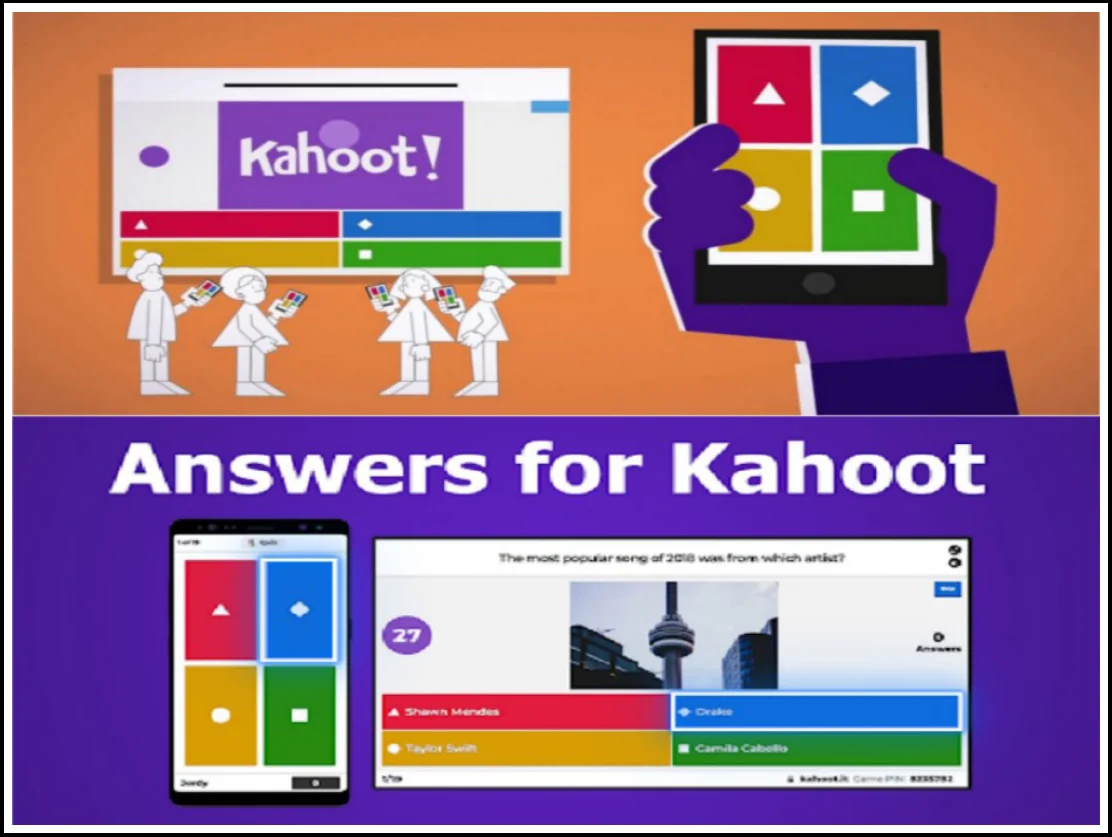
Kahoot Game based Quiz as an innovative Digital Tool for Learning.

## METHODOLOGY

A literature review was performed using such academic databases as PubMed, Scopus, Web of Science, and Google Scholar. The articles published between 2020 to 2025 were included in this review. The keywords utilized during the search process included: Kahoot, gamification, game-based learning, dental training and student involvement.

### Inclusion Criteria:

The study inclusion criteria were as follows:

- The study must have been testing Kahoot or gamification in dental or health professions education.
- The study must have been published in peer-reviewed journals.
- The study must have reported the outcomes of student engagement, learning outcomes, or knowledge retention.

### Exclusion Criteria:

- Non-academic reports.
- Articles on subjects not related to educational practice.
- Articles that could not provide their full text were excluded.

### PRISMA Flow description:

A total of 312 articles were obtained from different databases out of which 52 were duplicates. Remaining 260 records were screened and 210 records were again excluded from this review. The remaining 50 identified articles were found eligible to be included in this study while 20 full-text articles were excluded. Thus, total 30 articles were included in the qualitative synthesis as illustrated in the Fig.2 of PRISMA Flow Diagram.

**Fig.2:**
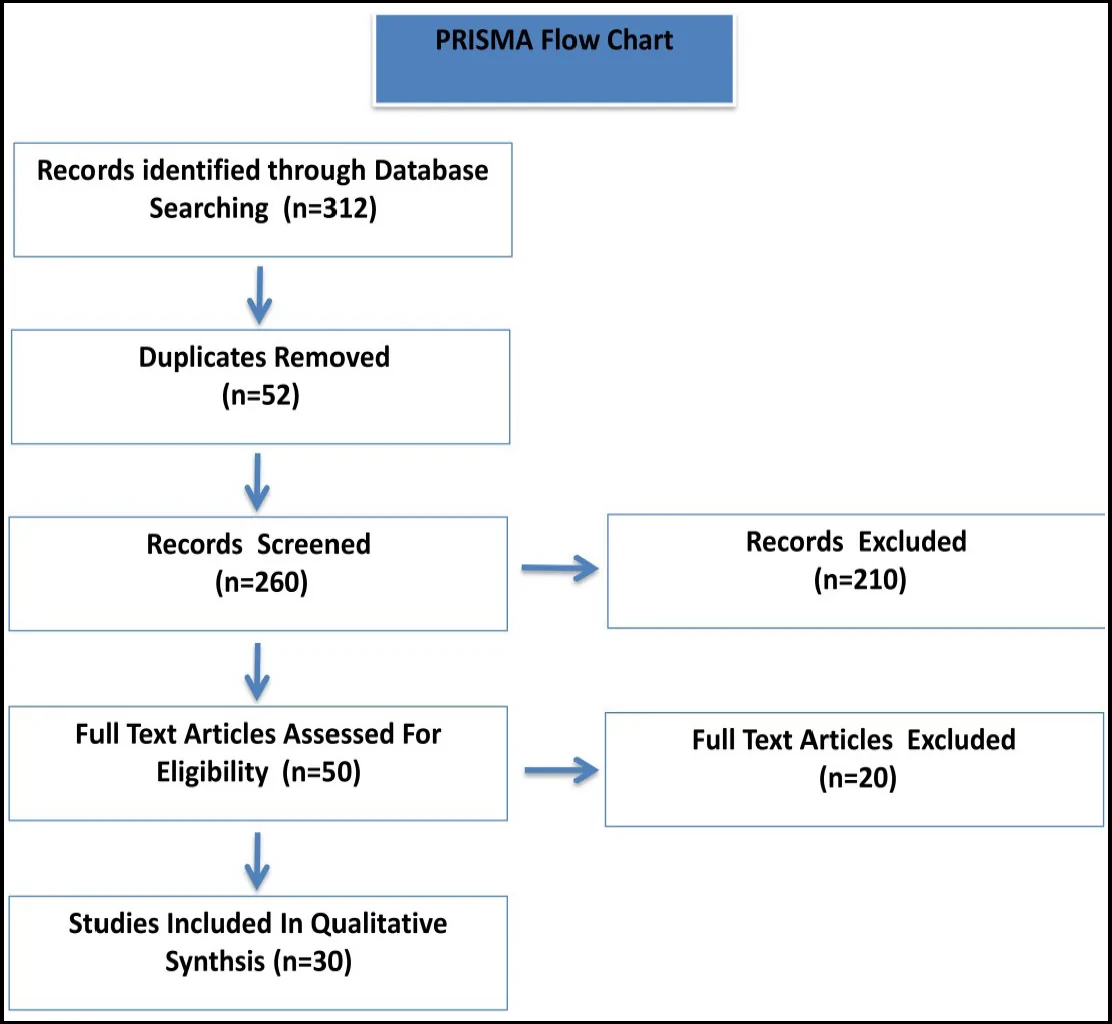
PRISMA flow diagram illustrating the study selection process for the narrative review on the use of Kahoot in dental education.

### Student engagement after using Kahoot:

Student’s active engagement is a factor that defines successful learning outcomes. Gamification tools like Kahoot also promote engagement of the learner by making lectures more interactive. The empirical research continuously confirms that Kahoot improves classroom interaction and focus in the students.^1^,^4^ This is because of the competitive system of the platform that compels the learners to respond to questions with great enthusiasm and keep track of their performance in comparison to the learners that gain knowledge through other sources. The dental students tend to state that Kahoot sessions are quite entertaining as well as helpful in maintaining attention during the lectures.^10^ Moreover, visual and auditory features of the Kahoot platform are also capable of enhancing the process of learning.

### Impact on Learning Outcomes:

Many studies have concluded that the academic performance of learners can be enhanced by means of gamification learning tools. A comparative study between traditional lectures versus Kahoot mediated sessions reported that learners who have been subjected to gamification quiz performed better as compared to the traditional teaching.^11^ Gamification quiz also allows the better retention of the information in less time, thus enhancing the memory and allowing the better understanding of the subject matter. The instant feedback of Kahoot quiz is also helpful to the students in identifying the misconceptions and rectifying the mistakes in time.^12^

### Knowledge Retention:

The retention of the attained knowledge is one of the major goals of the dental education. Gamification quizzes are an effective tool of the retrieval practice, a mental exercise that strengthens the long-term memory by remembering the material being studied.^13^ Students who use the Kahoot activities usually show better memory of lecture material during examinations. This enhancement is likely to be obtained as a result of the interactive quality of the Kahoot quiz that plays an important role in supporting the knowledge retention through consecutive questions in the form of active Quiz.

### Kahoot as Formative Assessment Software:

Formative assessment represents a mandatory part of the modern pedagogical practice. Kahoot allows the instructor to understand the level of student’s comprehension in real time and to modify teaching strategies to a great extent. Educators will be able to identify the areas of weakness and give specific clarification with immediate feedback and graphics showing the immediate response which is the trademark of Kahoot quiz.^14^,^15^

### Flexibility and Accessibility:

The ability to use Kahoot in a classroom or in a virtual environment highlights the flexibility of this tool revealing that it can be used on a variety of different devices that can possibly enhance its application in blended learning. Teachers implemented tools like Kahoot to maintain the interaction and flow of education during the COVID-19 pandemic in an excellent manner. This supports the usefulness of online, game-based quiz format of Kahoot in both classrooms and distance learning settings eventually, boosting its significance in blended curricula.^16^

### Collaborative Learning:

Kahoot also encourages collaborative learning as it allows the learners to work together in answering the quiz questions. The empirical data supports the idea that Kahoot will create a sense of a community and collaboration in learners and adds the depth to the educational experience. This goes in line with the social interaction factor that is imperative to blended learning and assists in the development of communication and teamwork skills.^17^

### Active Learning and Review:

Kahoot is a transformation of the passive revision into an active learning process. One of the research concluded that students who seriously learned the content of the curriculum by using game-based quizzes in Kahoot took a more active part, which resulted in the improved comprehension and knowledge of the material and consequently higher scores in the formative assessments. Kahoot can also serve the purpose of a formative evaluation tool where a teacher can determine certain areas of content where the learners may be having difficulties and therefore, can take immediate action and make corrective actions.^8^ This increased the importance of Kahoot as an aid of learning in the Learner Centered Approach learning style that can help learners to perform better in test, especially quizzes, instead of using only formal assessment that is carried out through the system. As a result, benefits of enhanced student interaction, high motivation rates, immediate feedback, active learning process, and enhanced retention of knowledge make Kahoot a worthy addition to the list of instructional tools in dentistry (Table-I).

**Table-I:** Summary of Selected Studies on Kahoot in Dental Education.

S.No.	Themes	Evidence Summary	Educational Implications
	Student	- Increased classroom participation	Kahoot transforms passive lectures into interactive learning with increased student involvement
	Engagement^1^,^4^,^5^,^9^,^22^	- Enhanced attention & enthusiasm	
		- Learning more enjoyable and interactive.	
	Learning Outcomes / Academic Performance^10^,^11^,^26^,^31^	- High post-test scores with Kahoot based learning in comparison with traditional methods	Kahoot reinforces lecture content with improved academic performance
	Knowledge	- Enhanced memory consolidation	Kahoot serve as an effective revision tool
	Retention^3^,^15^,^16^,^21^	- Long-term retention	
	Formative Assessment^8^,^17^,^18^,^25^	- Immediate feedback allows to identify knowledge gaps and clarify misconceptions in real time	Kahoot can be integrated as formative assessment strategy
	Student Motivation & Satisfaction^7^,^12^,^23^,^24^	- High motivation & enjoyment - Increased satisfaction levels	Kahoot intimates Positive learning and Encourages active learning
	Collaborative & Interactive Learning^16^,^19^,^28^,^29^	- Encourages peer discussion	Kahoot strengthen communication and teamwork skills among dental students
		- Increase teamwork	
		- More collaborative problem-solving activities	
	Technology Enhanced Learning in Dentistry^9^,^20^,^25^	- Integration of interactive teaching & learning	Kahoot Supports modern competency based dental education
	Challenges & Limitations^2^,^14^,^17^	- Excessive competition	Kahoot focuses on Applied and integrated teaching strategy
		- Technological dependence	
		- Lack of long-term retention	

## DISCUSSION

The novelty of this narrative review highlights the fast development of digitally operated educational technologies such as Kahoot quiz that has significantly changed the way pedagogy is provided in health professions education especially dentistry. The strength of this review article displays the help it provides to the learners in absorbing multiple theoretical knowledge and polishing clinical skills altogether with new digitalized teaching methods e.g. Kahoot quiz that has become a necessity. Gamification has presented itself as an interesting pedagogical tool for enhancing understanding through the incorporation of latest technology in the learning environment.

Kahoot! is one of the most important solution that gained popularity among the multitude of various educational platforms due to its interactive feature, easy-to-use interface with the ability of developing active interaction with learners. This narrative review reveals that Kahoot! is one of the most important tool that has greatly influenced the student engagement thus, improving their learning and knowledge retention in the field of dental education.^12^-^14^ Several studies have concluded that gamified learning tools like Kahoot trigger curiosity and motivation of learners as a result of their true competition, instant feedback and rewarding capabilities. These elements have been aligned with famous educational theories referred as self-determination theory and constructivist learning theory where learners have strong control, good efficacy and outstanding engagement in knowledge construction process.^12^-^14^

On the other hand, conventional teaching results in passive learning where students are only the information receivers while on line quiz turns a lecture into an interactive session where learners participate actively in positive real-time competitions with their colleagues.^1^,^2^,^15^ This real time engagement and long attention of learners using Kahoot quiz have been reported by the post-secondary institutions which in itself is an achievement at higher education level.^1^,^2^ It has also been observed these digital tools effectively magnify the learning process because of their entertaining nature hence improving the concentration and attention of students in the dental education.^5^

Kahoot! has brought remarkable revolution in the academic performance of the students at all the levels. Certain comparative studies confirmed that students who have been subjected to Kahoot! sessions displayed high test scoring in comparison to regular conventional approaches thus, showing that gamification amplified post-test scores.^18^-^20^ These studies indicate that game quizzes not only improve knowledge acquisition and learning retention but also the student’s engagement in the learning process that might be helpful in developing their grip on the subject. Hence, Kahoot! Is a proven tool in improving the academic performance because of its magnificent retrieval practice mechanism which is a cognitive process and plays an important role in strengthening the memory through repetition.^6^,^21^,^22^ Moreover, students get access to the new information and its retention in a very short time span which boots their long-term memory formation and is encouraging for attaining high academic grades.^6^,^21^,^22^

Kahoot! can also be used as a detecting resource as well to find out whether learners have perceived the entire knowledge information correctly or they need more knowledge transfer before their summative assessments. Although, Kahoot! is a formative assessment tool as it offers feedback to both learners and educators on a constant basis but it plays a very crucial role in the summative assessments also.^11^,^23^,^24^ Additionally, Kahoot has the capability of observing the errors and rectifying them instantly without a delay by providing a real time online feedback. Various studies have indicated that this timely feedback is improving the ability of students not only for assessing their learning progress but also modifying their educational strategies respectively.^11^,^23^,^24^ Kahoot also has an impressive advantage in facilitating group learning by promoting a teamwork that improves the peer interaction, communication and better understanding of complex issues.^25^,^26^ These collaborative activities enhance both the cognitive and interpersonal skills that promotes the clinical problem-solving and teamwork which is paramount in this digital era .

However, there are some disadvantages of Kahoot quiz that should be given great attention.^12^,^27^ One of the biggest problems related to use of this tool is the possibility of excessive focus on competition that would turn motivation into excessive anxiety hence, activating the learners in losing their concentration in education. Therefore, teachers are required to plan these competitive game activities in a way that they become productive in upgrading the learning process in a positive manner.^12^,^27^ Other possible disadvantage of Kahoot is the dependency on digital technology that needs constant internet connectivity and technological gadgets such as smartphones, tablets, computers or laptops.^28^ That might not be possible in developing countries like Pakistan where resources are very few and lack of proper technological infrastructure may be a barrier to the high application of such latest learning platforms. Moreover, the technical problems during the classes may interrupt the teaching process and reduce the success of these technologybased strategies.^29^,^30^

Furthermore, literature also reveals that there is no long-term evidence of knowledge retention and score improvement with these technology based tools 31,32 that needs to be investigated and addressed.^33^,^34^ Future studies are recommended to evaluate the effect of Kahoot in long-term knowledge retention, clinical competency check and summative assessment scoring improvement among dental students.^21^,^33^,^34^ The research findings on Kahoot quiz in dental education is compromised as a result of small sample size and single institution studies that decrease the extrapolability of findings and stress the necessity of multi-center research with a variety of students.^33^ The next generation of studies must apply a more powerful experimental design such as randomized controlled trials in producing more robust evidence regarding the educational efficiency of gamification in dentistry.^34^ More researches are required where Kahoot can be incorporated into blended learning and flipped classroom models where students should go through learning materials before lesson followed by interactive problem-solving on line technology based sessions.^31^ Kahoot could be an effective way of evaluating the pre-class and post-class preparation that would emphasize on the main learning concepts in the classroom which would play a crucial role in enhancing the performance levels of students to great extent.^31^ Kahoot has also not yet been investigated in terms of its potential application in clinical training but only on the theoretical classroom learning.^35^-^37^ It may be used to assist in clinical decision-making and diagnostic reasoning exercises where teachers can develop case-based Kahoot quizzes for embedding the theoretical material in practical cases. These techniques can develop the critical thinking skills and prepare students to work in the real-world clinical settings of the dentistry.^35^-^37^

Furthermore, artificial intelligence and educational analytics advancement may expand the learning systems that could evaluate the patterns of responses among students by providing personalized learning recommendations.^38^-^40^ The combination of gamification and adaptive learning technologies might help the facilitators to individualize teaching for addressing the learning needs of each learner thus, enhancing the educational outcomes.^38^-^40^ Additionally, faculty development is also crucial in the successful execution of such modernized teaching techniques because of non-availability of such tools or unwillingness for adopting such new technologies . As a result, it is advisable to develop training programs that enables faculty to design these effective learning activities within the existing curricula.

It is also essential to realize that Kahoot might not substitute the traditional teaching approach but could be used as an additional educational tool for successful dental education with a well-balanced combination of lectures, clinical training, problem-based learning and digital technology based innovations. Kahoot can complement these techniques in a prudent way such as: increasing motivation, strengthening knowledge and providing meaningful opportunities of education. The current literature suggests that Kahoot is a robust technology oriented platform that is capable of increasing student engagement, improving student grades and supporting knowledge retention in dental education. Nonetheless, further studies are required to overcome the existing methodological challenges and explore the educational implications of gamification in dental education especially in the long term. Future studies must focus on the multi-institutional study designs combined with the novel teaching models such as the flipped classrooms and the evaluation of clinical competency outcomes. Fusing the conventional approach to learning with the new online digital technologies, dental educators can be able to provide an exciting learning atmosphere that will equip the student more to the needs of the contemporary dental practice.

### Limitations

A number of constraints have been noted including: Firstly, gamified tools can only be implemented effectively when there is reliable broadband internet connectivity and relevant digital devices.^15^ Such prerequisites represent a serious impediment in an environment where the resources are limited. Secondly, over-reliance on gamification may indeed have the negative effect of derailing learners off the goals of core curriculum provision unless carefully monitored.^16^ Lastly, the majority of Kahoot research studies focus on short-term learning effects where future studies are needed to determine knowledge retention and clinical competence in the long term altogether with the summative assessment.

## CONCLUSION

Gamification is also a powerful pedagogical intervention that has the ability to revolutionize the traditional dental education. In this context, it has been found that Kahoot has produced a significant effectiveness in provoking the student activity, improving the learning results and facilitating the formative evaluation. Even though there is evidence available that substantially supports the advantages of education, additional empirical research is needed to assess long-term impacts as well as to enhance the implementation of gamification in dental programs.

## Recommendations

Future researches are needed for incorporating Kahoot into blended learning and flipped-classrooms where combination of gamification and other interactive pedagogies can contribute to the overall improvement of learners engagement and educational achievement. In addition, emerging technologies such as artificial intelligence and learning analytics can be promising in enhancing the efficiency of digital technology based learning platforms.
